# StressNet: a spatial-spectral-temporal deformable attention-based framework for water stress classification in maize

**DOI:** 10.3389/fpls.2023.1241921

**Published:** 2023-11-28

**Authors:** Tejasri Nampally, Kshitiz Kumar, Soumyajit Chatterjee, Rajalakshmi Pachamuthu, Balaji Naik, Uday B. Desai

**Affiliations:** ^1^ Department of Artificial Intelligence, Indian Institute of Technology (IIT) Hyderabad, Hyderabad, India; ^2^ Department of Electrical Engineering, Indian Institute of Technology (IIT) Hyderabad, Hyderabad, India; ^3^ Department of Agronomy, Professor Jayashankar Telangana State Agricultural University (PJTSAU), Hyderabad, India

**Keywords:** multispectral, multitemporal, UAV, stress classification, maize, BiLSTM, attention-based network

## Abstract

In recent years, monitoring the health of crops has been greatly aided by deploying highthroughput crop monitoring techniques that integrate remotely captured imagery and deep learning techniques. Most methods rely mainly on the visible spectrum for analyzing the abiotic stress, such as water deficiency in crops. In this study, we carry out experiments on maize crop in a controlled environment of different water treatments. We make use of a multispectral camera mounted on an Unmanned Aerial Vehicle for collecting the data from the tillering stage to the heading stage of the crop. A pre-processing pipeline, followed by the extraction of the Region of Interest from orthomosaic is explained. We propose a model based on a Convolution Neural Network, added with a deformable convolutional layer in order to learn and extract rich spatial and spectral features. These features are further fed to a weighted Attention-based Bi-Directional Long Short-Term Memory network to process the sequential dependency between temporal features. Finally, the water stress category is predicted using the aggregated Spatial-Spectral-Temporal Characteristics. The addition of multispectral, multi-temporal imagery significantly improved accuracy when compared with mono-temporal classification. By incorporating a deformable convolutional layer and Bi-Directional Long Short-Term Memory network with weighted attention, our proposed model achieved best accuracy of 91.30% with a precision of 0.8888 and a recall of 0.8857. The results indicate that multispectral, multi-temporal imagery is a valuable tool for extracting and aggregating discriminative spatial-spectral-temporal characteristics for water stress classification.

## 1 Introduction

The growth and health of the crop depend on several essential agronomic inputs Boyer (1982) such as water and soil nutrients like nitrogen and phosphorous. These factors play a pivotal role in determining both the quantity and quality of production. Water aids in the transportation of nutrients Gonzalez-Dugo et al. (2010) from the soil to different regions of the plant. Inadequate water supply leads to the development of abiotic stress in plants, disrupting their capacity Wang et al. (2016); Vicente et al. (2018) to carry out vital processes such as photosynthesis, affecting the crop’s yield. In the recent past, the phenomenon of global warming Mueller et al. (2012); Food and of the United Nations (2019) resulted in irregular rainfall patterns leading to water scarcity. Water shortage leads to diverse physiological changes, including loss of greenness and reduced leaf surface and biomass. Maize is a staple food around the globe and accounts for 36% of the world’s grain production, constituting nearly 9% of the Indian food basket Dataset IIMR (2020). Since there are about one to two kernels per plant, drought stress impacts Zhou et al. (2020); Liu et al. (2020) the quality, harvesting ability, and crop yield. As per the recent study by Laborde et al. (2020), the pandemic in 2019 (COVID) resulted in uncertainties in global food security. Owing to the potential that maize occupies a significant amount towards ensuring the food supply, especially in developing nations like India, it is necessary to advance crop monitoring methods through comprehensive geographical evaluation. Accurate determination of optimal timing and quantity of water will facilitate enhanced irrigation.

Over the last decade, remote sensing methods have been extensively used by Semmens et al. (2016); Thorp et al. (2018); Tian et al. (2020) for characterizing water stress in crops. Aerial-based remote sensing emerged as a non-invasive technique to gather data from crop, soil, and environmental factors. It made a significant impact by obtaining “farm” level to “leaf” level information through image data. Further, this data helped Berni et al. (2009); Al-Tamimi et al. (2022) in quantifying various traits of water stress responses. Of the current aerial remote sensing techniques, Unmanned Aerial Vehicles (UAVs) have surfaced as efficient platforms for high-throughput phenotyping to monitor crop fields due to their high spatial and temporal resolution, further resulting in the improvement of the management of water stress in agriculture. UAVs can be accommodated with different types of camera sensors. They can fly at lower altitudes, cost-effective, enabling increased monitoring frequencies Berni et al. (2009); Araus and Cairns (2014); Gago et al. (2015).

Over the recent years in the field of computer vision, from conventional image processing techniques to present novel methods, automated learning-based feature extraction techniques have made substantial progress Li et al. (2020). These popular techniques include Support Vector Machine, K-Means clustering, and Random Forest. Moreover, Deep Learning (DL), a method that leverages LeCun et al. (2015) hierarchical feature extraction from images, has opened up new possibilities for interpreting vast amounts of data and permeated the field of data analytics in the field of agriculture. The plant science community is increasingly embracing these DL methods to extract meaningful insights from the extensive datasets gathered through high-throughput phenotyping and genotyping methods Kamilaris and Prenafeta-Boldú (2018); Zhong et al. (2019); Wang et al. (2022). Convolutional Neural Networks (CNNs) have gained popularity among Deep Learning methods for their ability to automatically extract valuable information from diverse features such as colour, shape, texture, size, and spectral information across different levels without the need for human expertise Krizhevsky et al. (2012); Grinblat et al. (2016); Lee et al. (2017). The exhaustive review from Singh et al. (2018) offers a thorough evaluation of DL methods applied to a broad spectrum of plant species, focusing on tasks such as identifying, classifying, quantifying, and predicting plant stress. The other studies of Kumar et al. (2020); Tejasri et al. (2022) explored UAV-captured imagery for predicting water stress-affected crops using CNN-based frameworks. These studies highlight that Red, Green, and Blue (RGB) bands are crucial for classifying water-stressed crops due to their rich properties of colour and texture. However, RGB bands are particularly light-sensitive and can only provide details within the visible spectrum Nijland et al. (2014). Moreover, multispectral data is of paramount importance due to its additional spectral information greatly aided Zarco-Tejada et al. (2012); Nijland et al. (2014); Wang et al. (2022) to overcome the light sensitivity issues in the visible spectral domain and helps in identifying the underlying information on crop water stress.

Earlier studies by Spišić et al. (2022); Barradas et al. (2021), utilized multispectral data and Supervised Machine Learning (ML) based methods to effectively detect drought stress in crops. These methods used MultiLayer Perceptron (MLP), Support Vector Machine (SVM), decision tree, Random Forest based classifiers, and gradient boosting techniques to classify water stressed plants. Virnodkar et al. (2020) conducted an extensive review on the use of supervised ML methods for crop water stress classification using UAV captured multispectral imagery. However, these described methods are mainly limited to manual feature extraction and thus are inefficient, particularly when dealing with high dimensional data or in complex environments Wang et al. (2022); Bouguettaya et al. (2022). This inherent limitation of traditional machine learning techniques has prompted a shift in focus towards machine learning methods based on DL LeCun et al. (2015).

By leveraging DL techniques with multispectral data, a significant transformation is occurring within the domain of data-centric agriculture. While CNNs show promising results in water stress detection and classification, as demonstrated by Kumar et al. (2020), they do not take temporal data into account. CNNs are limited by the assumption that data captured at different time points are equivalent. However, it is well-known that visual changes resulting from water stress in crop occur gradually and are not immediately discernible. This poses a challenge for CNNs, as they lack the ability to effectively learn temporal patterns, resulting in difficulties in confidently classifying stress conditions, as discussed by Singh et al. (2018); Gao et al. (2020). Moreover, the time-invariant nature of CNNs requires data displaying severe signs of stress for reliable detection, making it impractical for early identification and recovery of stressed plants. Therefore, there is an increasing need for a technique capable of analyzing the progressive visual changes in stressed plants, enabling confident classification even in the absence of severe stress signs, facilitating early-stage water stress classification, and addressing a critical gap in current methods. In this context, Elsherbiny et al. (2022) explored a CNN-LSTM approach to assess the water status of wheat. This study aggregated features derived from RGB images, climatic conditions, and soil moisture, achieving a remarkably low loss of 0.0012. In our preliminary study Tejasri et al. (2023), we utilized CNNs (AlexNet, VGG-19, ResNet18, ResNet-50) for extracting the features from multi-temporal multispectral UAV-captured maize data. The extracted visual features are further fed to a single LSTM unit for capturing temporal dependencies. The results showed that the model based on fine-tuned ResNet-18 backbone, using multispectral data outperformed with a precision of 0.9765 and a recall of 0.9457 rather than just using RGB data with a precision of 0.9523 and a recall of 0.9487. On the other hand, considering the change in environment and the crop conditions, this analysis becomes difficult with the help of a single LSTM unit.

Thus, a series of LSTM units can be made use of where the input to these units are the sequences of visual features that are extracted by CNNs to preserve the temporal patterns as demonstrated by Azimi et al. (2021), for identifying water stress in chickpea plant. This approach gained more insights by providing a more accurate representation of the relationship between the environmental conditions and the crop’s response. The sampling positions of standard convolution kernels remain constant. They cannot be adjusted to accommodate intricate spatial patterns in crop classification, as noted by Feng et al. (2020) in their work on multispectral image analysis. In addition, the classic pooling layers (average or max pooling) are also fixed and do not possess the capability to learn the downsampled features. Conversely, deformable convolution proposed by Dai et al. (2017), enables the neural network to adaptively adjust the sampling locations, allowing it to effectively capture the spatially varying patterns. Deformable convolution is an extension of standard CNN by introducing learnable offsets to the standard grid sampling locations of convolution kernels. Studies by Zhu et al. (2018) explored a deformable convolution neural network (DCNN) for hyperspectral image classification. Feng et al. (2020) adopted a deformable CNN-LSTM-based network for vegetable mapping from multi-temporal UAV-based RGB imagery. Motivated by the works mentioned above, we propose a model entitled StressNet which combines a deformable based CNN and a BiLSTM with weighted attention to dynamically adjust the receptive field to accommodate the size of the crop according to its growth stage.

In this study, we present a DL-based temporal analysis pipeline for classifying water-stressed crops, utilizing multispectral data captured by UAV. We aim to showcase the great performance of the proposed method compared to standard CNN, which is time-invariant and only spatial. The following contributions are obtained from the present work:

1. Dataset is created by using multispectral data of maize crop captured by UAV.
2. Our proposed model leverage the capabilities of CNN by adding deformable convolutional layer and BiLSTM for enhanced performance. It is specifically designed to learn spatial-spectral-temporal patterns for identifying water stressed crops.
3. We conducted a comparative analysis of the proposed method using CNN based architectures - AlexNet and VGG-16.
4. We performed an ablation study by evaluating the impact of temporal and spectral data using the proposed model. This involved systematically reducing the number of temporal data used and the number of spectral channels. In addition, we discussed the impact of the deformable convolutional layer, BiLSTM and weighted attention on the performance of the proposed method.

## 2 Materials and methods

### 2.1 Experimental site

**Figure 1:**
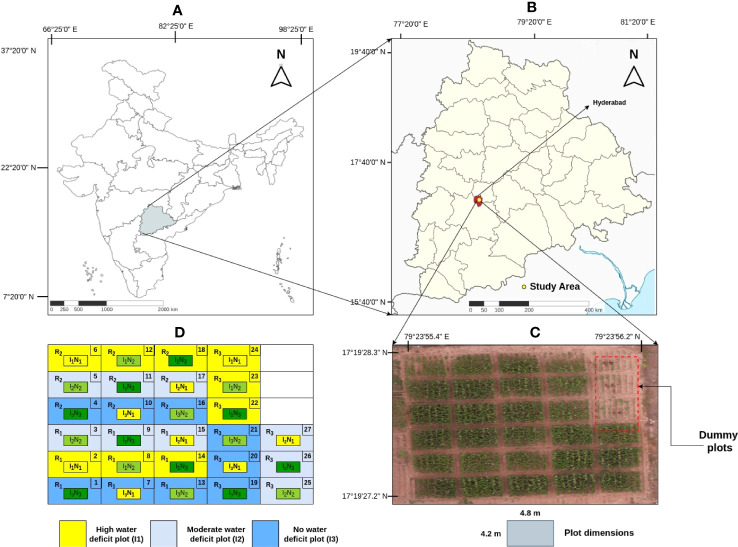
**(A)** Indian map. **(B)** Location of experiment field in Telangana map. **(C)** Top view of the field captured by the UAV with dummy plots highlighted. **(D)** Field layout of treatments where *I*_1_, *I*_2_, *I*_3_ represent high, moderate and no water deficit plots respectively.

The experimental study was conducted in a semi-arid zone of Hyderabad (Telangana, India) from October to February (post-monsoon season - Rabi) during 2018-19. The study area lies between 17°19’27.2”N – 17°19’28.3”N and 78°23’55.4”E – 78°23’56.2”E shown in **Figures 1A, B**. Rabi season was particularly chosen to precisely understand the water stress effect on the crop as the crop can be induced by heavy water stress conditions as the rainfall level is comparatively low during this period. The farm is situated in a semi-arid region, characterized by an average annual precipitation of 822 mm and annual potential evapotranspiration ranging from 1700 to 1960 mm. The soil in this area is predominantly composed of light red sandy loam and extends to a depth of approximately one meter and bedrock beneath it. For the study, maize crop (*Zea mays L.*) of the *‘Cargill 900 M Gold’* variety is cultivated. The farm was maintained by *Agro Climate Research Center, Professor Jayashankar Telangana State Agriculture University* (PJTSAU), Hyderabad, India. The experimental field comprises 30 regions, each measuring 4.2 m × 4.8 m. The experimental field was designed in a split mode with three irrigation and nitrogen supply levels based on a climatic approach Halagalimath et al. (2017).

The determination of the irrigation schedule was based on Reddy and Reddy (2019) the ratio of Irrigation Water (IW) to Cumulative Pan Evaporation(CPE). Three distinct irrigation levels are chosen, with IW/CPE ratios of 0.6, 0.8, and 1 assigned to the respective regions. For each irrigation event, a uniform quantity of 50 mm water (IW) is provided to the designated plots using pipes equipped with water meters to ensure accurate measurement. Pan evaporimeters (in mm) are used to record daily readings, aiding in the calculation of the IW/CPE ratio. This ratio was crucial in determining the ideal timing for irrigation across various regions. Additionally, each type of irrigation plot is subjected to one of three nitrogen fertilization levels: 100, 200, and 300 kg nitrogen per hectare, as represented in **Table 1**. By combining the three irrigation levels with the three fertilization levels, a total of nine distinct regions are created. Furthermore, each plot is replicated three times, resulting in a total of 27 plots (3 water levels × 3 nitrogen levels × 3 replications), as depicted in **Figure 1C**. In order to introduce diversity, each plot, that measures 4.2 m × 4.8 m, received one of three distinct combinations of water and nitrogen levels. This setup allowed for categorizing areas into conditions of low, moderate, and high water and fertilizer stress plots. In each plot within rows, the plants are spaced 20 cm apart from each other, and rows are spaced 60 cm apart for each treatment, resulting in an estimated plant density of 8.33 plants per square meter as shown in **Figure 1D**.

**Table 1:** Treatment information of the research farm for Rabi season (Winter 2018–19).

Treatment	Detail	Application Rate
$I_{1}$	High water stress	IW/CPE = 0.6
$I_{2}$	Moderate water stress	IW/CPE = 0.8
$I_{3}$	No water stress	IW/CPE = 1.2
$N_{1}$	High nitrogen stress	100 kg/ha
$N_{2}$	Optimum nitrogen	200 kg/ha
$N_{3}$	Overdose nitrogen	300 kg/ha

Here, IW means irrigated water in millimeter and CPE represents cumulative potential evaporation in mm. Nitrogen is supplied in kilogram per hectare (kg/ha).

### 2.2 Dataset collection

To ensure an accurate geo-referenced data acquisition, we deployed nine Ground Control Points (GCPs) that are surveyed using a Trimble R10 GNSS Receiver within the field. The images are captured using a DJI Inspire-1 Pro UAV equipped with a Micasense RedEdge-MX multispectral camera included with a Downwelling Light Sensor (DLS) (represented in **Supplementary Figure S1**). This sensor is a 5-band light sensor that calculates the surrounding light conditions during a flight for each of the camera’s five spectral bands and then stores this data within the metadata of the captured images. After calibration, this information is used to rectify the illumination changes in the middle of a flight that takes place due to cloud cover. Using Mission Planner version 4.3.1 (ArduPilot Dev team), the UAV flight path is predetermined at an altitude of 10 meters with a speed of 4 km/hr. The pixel resolution was set to 2 cm. Vertical overlap of 70-80% and horizontal overlap of 50-70% is maintained in consecutive images to ensure maximum coverage. The collected data consists of five spectral bands, blue (475 nm), green (560 nm), red (668 nm), red-edge (717 nm), and near-infrared (NIR) (842 nm) regions. In this study, crop cultivated from the tillering stage through the heading stage is considered. Radiometric calibration is carried out for the utilization of UAV-based multispectral imagery. It considers various factors, such as the position of the sensor and sun, camera gain, exposure information, and irradiance measurements that may affect the quality of image data. For radiometric calibration, images of the Calibrated Reflectance Panel (CRP) are captured by the camera and DL sensor before the UAV flight.

### 2.3 Data pre-processing

Each CRP is associated with a calibration curve spanning the visible and NIR spectrum. Absolute reflectance values in the range of 0 to 1 are related to the range of 400 - 850 nm (with a 1 nm increment). To perform radiometric calibration, the captured panel images are loaded with the above values provided by Micasense on Agisoft Metashape® Professional (Version 1.8.3 build 14331 64-bit) photogrammetry software. To obtain a complete field perspective, the raw photos are aligned, geo-rectified, and further stitched, based on similar image characteristics. After the alignment, the high-quality and mild filter mode options are used to create a dense point cloud. A Digital Elevation Model (DEM) and an orthomosaic (a panoramic picture stitched together and geometrically corrected) of each band, covered by the corresponding raw images, are exported (shown in **Supplementary Figure S2A**). The settings employed in the Agisoft Metashape software for the creation of orthomosaic are reported in **Table 2**. The shape files corresponding to orthomosaic are created using open source QGIS® tool, and using these files, subplot containing region of interest, are extracted using RStudio (shown in **Supplementary Figure S2B**). The net area is considered in the process to ensure that the impact of crops on the boundaries does not have any effect. This is obtained by removing 5% of the outer perimeter on each edge of the image. By performing the sliding window method on this extracted image, Region of Interest (ROI) of individual plants is extracted.

**Table 2:** The settings employed in the Agisoft Metashape software for the creation of orthomosaic.

Sparse point cloud	
Accuracy	Medium
Image pair selection	Ground control Point
Constrain features by mask	Exclude Stationary tie points
Maximum number of feature points	20,000
Dense point cloud	
Quality	Medium
Depth filtering	Mild
Digital Elevation Model(DEM)	
Type	Geographic
Coordinate system	WGS 84 (EPSG::4326)
Source data	Dense cloud
Orthomosaic	
Surface	DEM
Blending mode	Mosaic

### 2.4 Methodology

**Figure 2:**
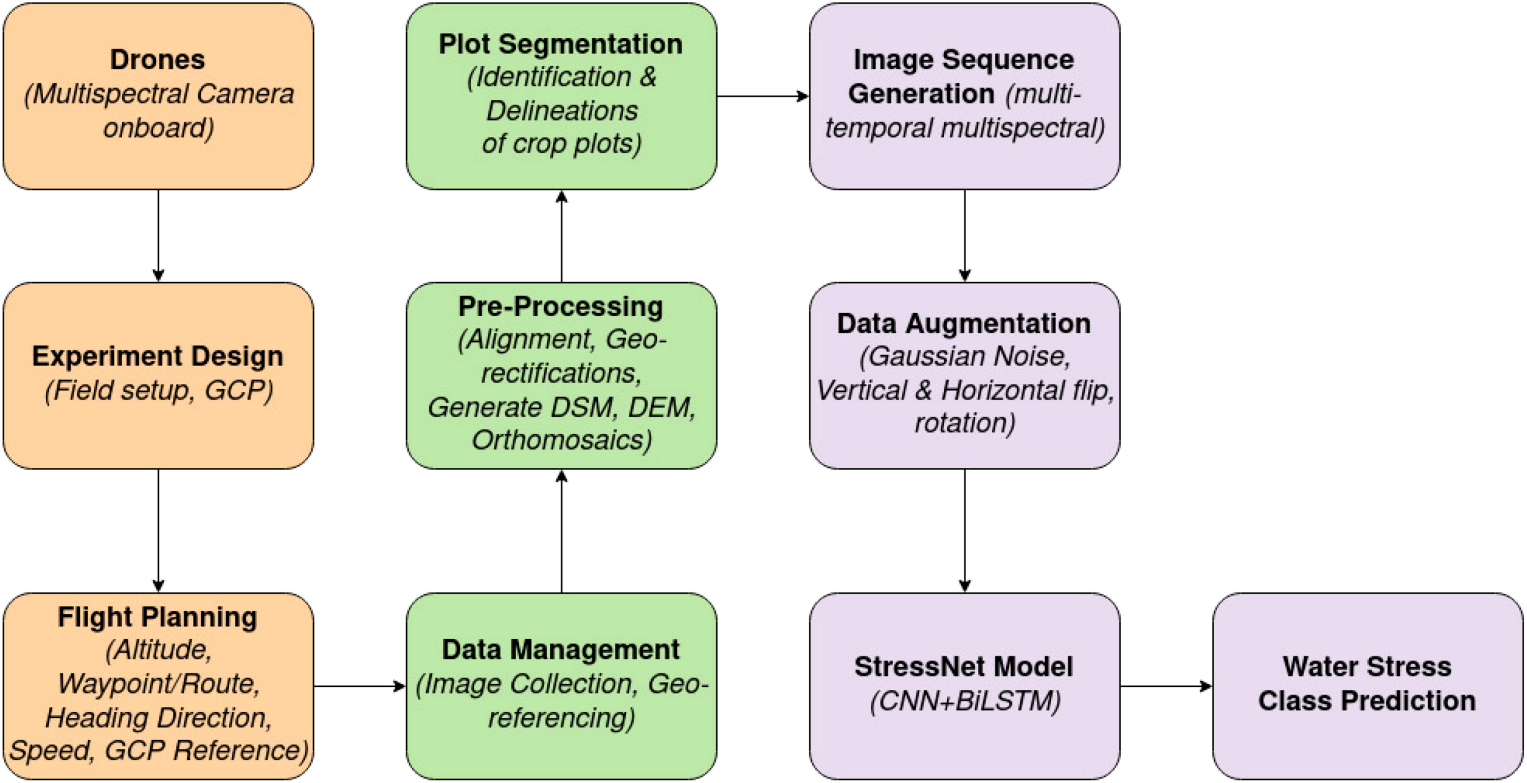
Our pipeline illustrates all the steps involved in water stress classification.

Our proposed framework’s workflow is illustrated in **Figure 2**, outlining all the steps undertaken in this study.

#### 2.4.1 Overview of StressNet

Convolutional Neural Networks (CNNs) can be divided into two main components. The initial component, often referred to as the ‘backbone,’ comprises a series of convolutional and pooling layers aimed at extracting intricate features. These layers function as feature detectors, sampling the input image data to produce high-level feature maps. In simpler terms, specific neurons within these layers become active when certain features are detected in the input image. While the initial layers are proficient at capturing basic features like edges, the deeper layers excel at identifying more complex characteristics, such as textures and the shapes of specific objects. The second component, known as the ‘head,’ learns from the extracted features and produces results tailored to the specific application Zeiler and Fergus (2014).

**Figure 3:**
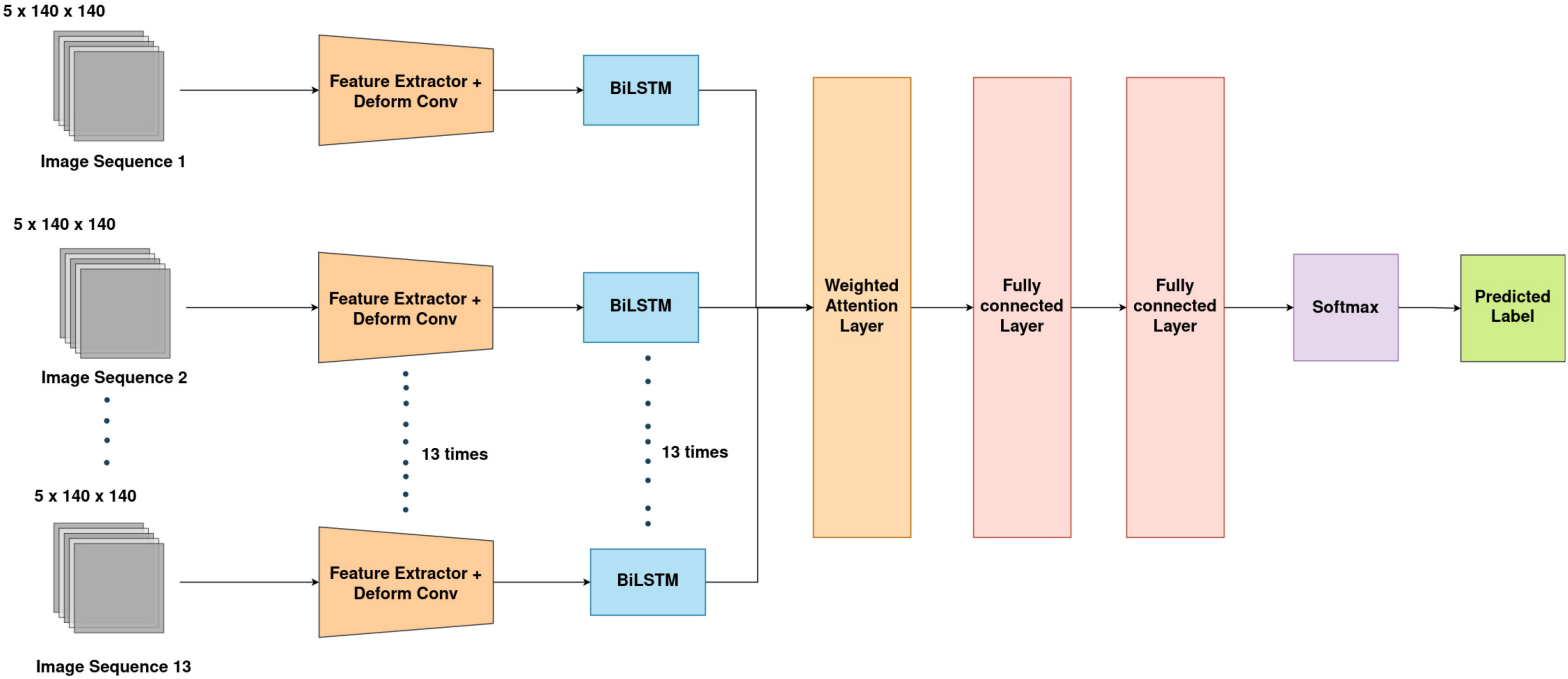
Overview of StressNet model. Input image sequence, Feature Extractor, Sequence processing BiLSTM network and Weighted attention modules are shown.

As for the proposed model, StressNet, it comprises two key components. The first is a feature extraction module based on a CNN, while the second is a spatial-spectral-temporal feature fusion module using BiLSTM network and an attention mechanism. The feature extractor module captures spatial features across multiple spectral channels. These spatial-spectral and temporal features are then aggregated using the BiLSTM network and a weighted attention mechanism to achieve the final water stress classification. The architecture of the proposed model is depicted in **Figure 3**.

#### 2.4.2 Spatial-spectral feature extraction

The input for the feature extractor is in the form of *k* x *k* x *c*, where *k* x *k* represents the patch size and *c* denotes the number of channels. The final convolutional layer of the backbone network is replaced with a deformable convolutional layer. Deformable convolution is an extension of standard convolution that introduces additional parameters to control the sampling locations within the receptive field. Unlike the standard convolution, where the sampling grid is fixed, deformable convolution enables the network to learn spatial transformations and adapt its sampling locations dynamically Dai et al. (2017); Jin et al. (2019). The continuous increase in water stress leads to physiological changes in the crop, such as a decrease in the surface area of the leaf, which further leads to the twisting and rolling of the leaf Spišić et al. (2022). Deformable convolution enables the kernel to adjust its receptive field to the target size of the crop according to its growth stage and water stress condition with additional offsets. These offsets are updated during the training phase of the model Dai et al. (2017). Equation 1 is used for determining the output *y* at the location *a*_0_, where *x* represents the input feature map, *w* stands for the learned weights, *a_i_* specifies the *i_th_* location and Δ*a_i_* denotes the offset to be learned.


(1) $$y\left(a_{0}\right)=\sum^{\;}w\left(a_{i}\right)\;*\;x\left(a_{0}+a_{i}+\text{Δ}a_{i}\right)$$

#### 2.4.3 Spatial-spectral-temporal feature fusion

**Figure 4:**
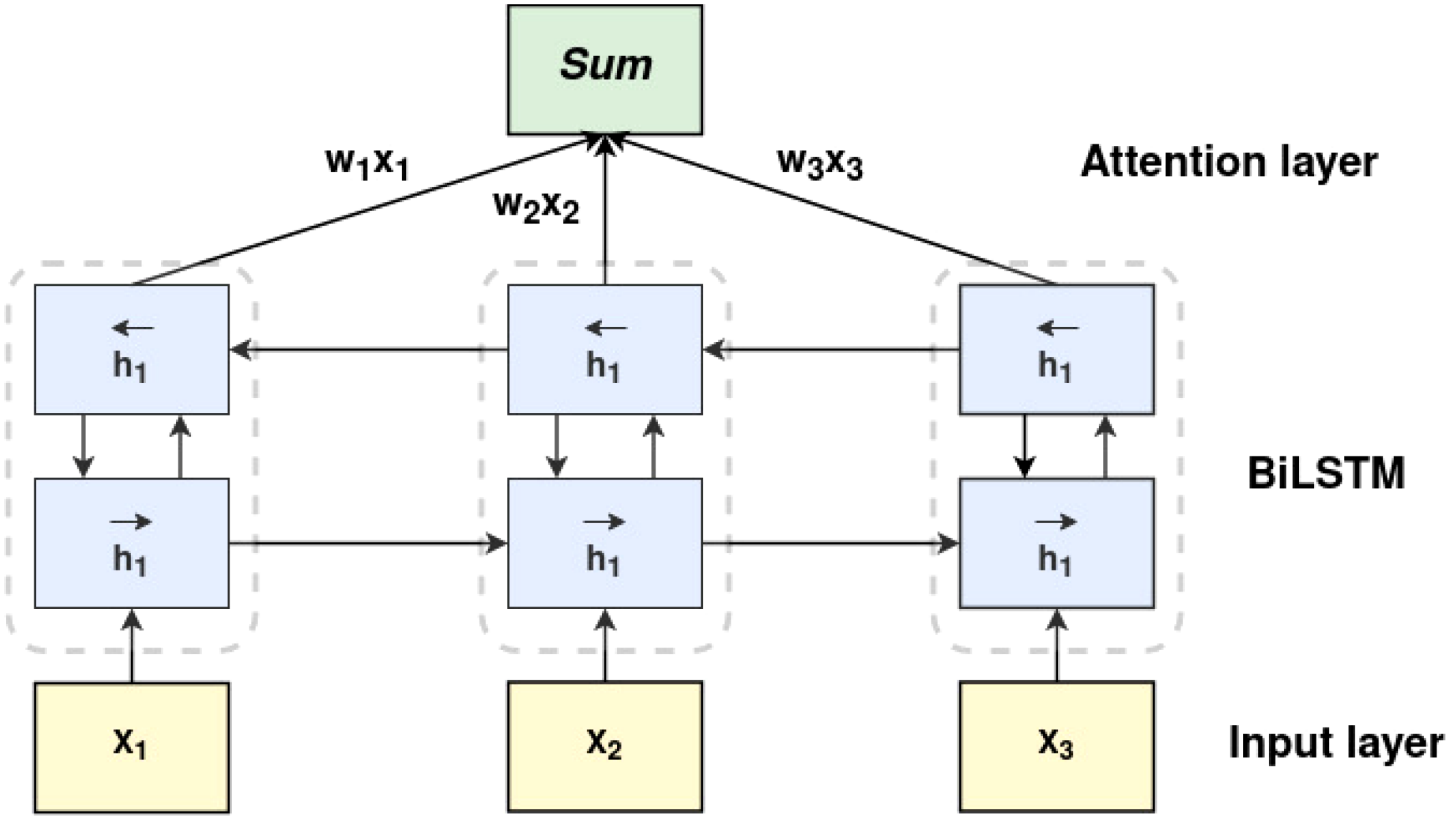
The architecture of the weighted attention-based bi-directional LSTM. *x*_1_, *x*_2_, *x*_3_ correspond to features obtained by the feature extractor. *h*_1_ typically refers to the hidden state output of the forward LSTM layer.

After extracting spatial and spectral features by deformable-based CNN, it is essential to capture the relationship between the temporal dependencies within the features. To achieve this, a BiLSTM network similar to that of Melamud et al. (2016) is employed. A BiLSTM layer is added to each feature extractor. The output of each feature extractor is given to the BiLSTM layer. Each BiLSTM is stacked with two LSTM layers, where the hidden state of the first LSTM is an input for the second LSTM, illustrated in **Figure 4**. By processing the sequential signals in reverse order, the second LSTM layer enables a detailed understanding of the inter-dependencies within the data.

Equation 2 computes the input gate’s output, determining how much of the new input shall be stored in the cell state *c_t_*. On the other hand, Equation 3 corresponds to the forget gate *f_t_*, which decides how much of the input *x_t_* and previous cell state *h_t_*_−1_ is to be retained for the current time step. Further, Equation 4 updates the cell state *c_t_* by removing some information based on the forget gate *f_t_* and adding new information scaled by the input gate *i_t_*. Equation 5 denotes the output gate that determines how much of the cell state’s information should be passed to the hidden state. Finally, Equation 6 computes the new hidden state based on the cell state and the output gate’s decision. In summary, these equations represent the working of an LSTM cell that helps the network learn and store information over longer sequences by controlling the flow through the cell state and hidden state using gates.


(2) $$i_{t}=\sigma \;\left(W_{ix}x_{\text{t}}+W_{ih}h_{t-1}+b_{i}\right)$$

(3) $$f_{t}=\sigma \;\left(W_{fx}x_{t}+W_{fh}h_{t-1}+b_{f}\right)$$

(4) $$c_{t}=f_{t}c_{\text{t}-1}+i_{t}\tanh\;\left(W_{cx}x_{\text{t}}+W_{ch}h_{t-1}+b_{c}\right)$$

(5) $$o_{t}=\sigma \;\left(W_{ox}x_{t}+W_{oh}h_{\text{t}-1}+b_{0}\right)$$

(6) $$h_{t}=o_{t}\tanh\;\left(c_{t}\right)$$

where, *i* refers to the input gate, *f* stands for the forget gate, *o* refers to the output gate, *c* is the memory cell and *σ* stands for the logistic sigmoid function.

To further improve the model’s performance, a weighted attention layer is applied to the outcome of the second LSTM. By assigning varying degrees of importance to different input features, the attention layer dynamically adjusts the weights according to the input feature so that the model focuses on the most pertinent information. Consider *H* to be a matrix that contains the BiLSTM’s output vectors [*h*_1_, *h*_2_,…, *h_T_*], where *T* stands for the length of the input features. The weighted sum of vectors adds up to the output of the attention layer and is described by the following equations 7, 8. The softmax activation function is a commonly used activation function in neural networks. It is used to transform the output of a neural network into a probability distribution. This transformation is defined by equation 9. Equation 10 refers to the ‘combined’ and ‘attention-weighted’ spatial-spectral-temporal features *R_att_*, where *α* represents the attention vector. The BiLSTM-Attention features undergo an adaptive re-weighting or re-calibration, enhancing the significance of valuable feature vectors and diminishing the unwanted or noisy ones. Subsequently, these re-weighted features are connected to two fully connected layers and a softmax classifier. The output of the softmax classifier is a vector of probabilities where each element corresponds to the probability of the input belonging to a specific class.


(7) M=tanh(H)

(8) $$\alpha =\text{softmax }\left(w^{T}M\right)$$

(9) $$\;\text{where},\;\text{softmax }\left(z_{j}\right)=\frac{e^{z_{j}}}{\sum_{k=1}^{K}e^{z_{k}}}\;\text{for}\;\text{j}=1,\ldots ,\text{K}$$

(10) $$R_{\text{att }}=H\alpha^{T}$$

#### 2.4.4 Data preparation

The training data is classified into three categories, namely, highly water-stressed, moderately waterstressed, and unaffected. Each class has 32 image sequences of 13 images of 5 channels. Each image has a dimension of 140 x 140 pixels. The Standard image normalization method is performed for all the channels by scaling all values to fit within the range of [0, 1] or adjusting the first- and second-order moments to achieve a mean of zero and a variance of one. All the channels of multispectral data are loaded into a sequence of the length of the days on which the data is captured using a custom data function. The ratio of training and validation is considered as 4:1.

#### 2.4.5 Training details

Popular CNN-based models such as AlexNet Krizhevsky et al. (2012) and VGG-16 Simonyan and Zisserman (2014) architectures are employed as backbones of feature extractor. The first layer of CNN of the proposed model is modified to work with input of 5 channels instead of 3. Detailed configuration of the feature extractor with AlexNet and VGG-16 are shared in **Tables 3**, **4**, respectively. During training, the model’s weights are initialized using He initialization He et al. (2015), and biases are set to zero. The categorical cross-entropy loss function CE, represented in equation 11, is employed to train our model. This loss function considers the one-hot representation of the ground-truth label *y*, the predicted outcome *y_p_*.

**Table 3:** Detailed configuration of the feature extractor with AlexNet backbone.

Layer Name	Input Size	Output size	Kernel Size	Padding	Stride
	(H x W x Channels)	(H x W x Channels)			
Input	140 × 140 × 5	–	–	–	–
Conv1	140 × 140 × 5	– × – × 96	11 × 11	0	4
Conv2	– × – × 96	– × – × 256	5 × 5	2	1
Conv3	– × – × 256	– × – × 384	3 × 3	1	1
Conv4	– × – × 384	– × – × 384	3 × 3	1	1
Deform Conv Layer	– × – × 384	4 x 4 x 256	3 x 3	1	1

H,W denotes height and width of input respectively. Conv stands for Convolution. Deform Conv stands for Deformable convolutional layer.

x - is understood as the output size of feature map after convolution operation.

**Table 4:** Detailed configuration of the feature extractor with VGG-16 backbone.

Layer Name	Input Size	Output size	Kernel Size	Padding	Stride
	(H x W x Channels)	(H x W x Channels)			
Input	140 × 140 × 5	–	–	–	–
Conv1	140 × 140 × 5	– × – × 64	3 × 3	1	1
Conv2	– × – × 64	– × – × 64	3 × 3	1	1
Conv3	– × – × 64	– × – × 128	3 × 3	1	1
Conv4	– × – × 128	– × – × 128	3 × 3	1	1
Conv5	– × – × 128	– × – × 256	3 × 3	1	1
Conv6	– × – × 256	– × – × 256	3 × 3	1	1
Conv7	– × – × 256	– × – × 256	3 × 3	1	1
Conv8	– × – × 256	– × – × 512	3 × 3	1	1
Conv9	– × – × 512	– × – × 512	3 × 3	1	1
Conv10	– × – × 512	– × – × 512	3 × 3	1	1
Conv11	– × – × 512	– × – × 512	3 × 3	1	1
Conv12	– × – × 512	– × – × 512	3 × 3	1	1
Deform Conv Layer	– × – × 512	4 x 4 x 512	3 x 3	1	1

H,W denotes height and width of input respectively. Conv stands for Convolution. Deform Conv stands for Deformable convolutional layer.


(11) $$CE=-\sum_{i}y_{i}^{p}\log\;\left(y_{i}\right)$$

A batch size of 16 is utilized, and the Adam optimizer proposed by Kingma (2014) is employed with a learning rate of 1e-4. To address the limited data in the study, data augmentation technique is used. This involved rotating all training images by 90 degrees and randomly flipping them horizontally and vertically. The model is built using the PyTorch framework, and the training process is executed on a computer running on the Ubuntu 20.04 operating system. The training is implemented on Intel(R) Xeon(R) Platinum 8168 CPU with 24 cores and an NVIDIA Tesla V100-SXM3 Graphics Processing Unit (GPU) with 32 GB RAM.

#### 2.4.6 Evaluation metrics

The assessment of the proposed model is conducted using the performance metrics that include Accuracy (Acc), Precision (Pre), and Sensitivity/Recall are defined in equations 12, 13, and 14 respectively. *FN* denotes False Negatives, *TN* corresponds to True Negatives, *TP* represents True Positives, and *FP* represents False Positives with respect to the actual and predicted water stress class.


(12) $$Accuracy=\frac{TP+TN}{\left(TP+TN+FP+FN\right)}$$

(13) $$Precision=\frac{TP}{\left(TP+FP\right)}$$

(14) $$Sensitivity/Recall=\frac{TP}{\left(TP+FN\right)}$$

## 3 Experiments and results

### 3.1 Results of the proposed model

**Figure 5:**
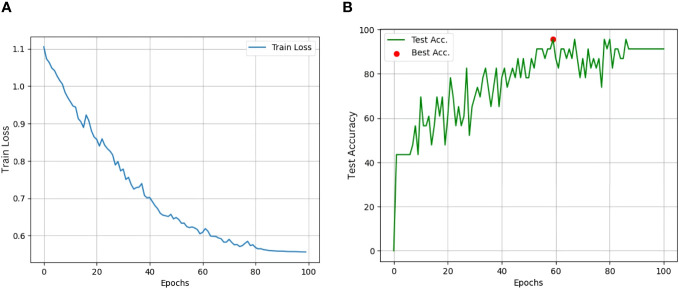
**(A)** Training loss of best StressNet model; **(B)** Validation Accuracy of best StressNet model.

We conducted spectral analysis and temporal analysis to highlight the efficiency of the proposed method. For the spectral analysis, we validated the model’s performance by considering all 13 days’ data of RGB channels or RGB with either NIR or red-edge channels. The results of spectral analysis are reported in **Table 5**. In the temporal analysis experiment, we assessed the model’s performance by gradually adding the data from 3 to 13 days by utilizing all spectral channels. The results of the temporal analysis experiment are reported in **Table 6**. It is observed that the proposed model with VGG-16 backbone achieved the highest validation accuracy of 91.30%, a precision of 0.8888, and a sensitivity of 0.8857 when using all five spectral channels and data collected for up to 13 days. The class-level accuracies and the classification report of the best model are reported in **Tables 7**, **8**, respectively. The training loss and validation accuracy graphs are represented in **Figures 5A, B** respectively.

**Table 5:** Spectral analysis of StressNet model with AlexNet and VGG-16 backbones.

No. of Channels		AlexNet				VGG-16		
	Tr. Loss	Val. Acc.	Pre	Se	Tr. Loss	Val. Acc	Pre	Se
RGB	0.5521	73.913	0.5694	0.5206	0.5523	65.2174	0.7833	0.4777
RGB-NIR	0.5519	86.9565	0.7606	0.6793	0.5516	82.6087	0.7575	0.5936
RGB-Re	0.5516	73.913	0.6613	0.6682	0.5517	82.6087	0.6666	0.6349
All	0.5619	82.6087	0.7888	0.7888	0.5515	91.3043	0.8888	0.8857

(Tr. Loss, Training loss; Val. Acc., Validation Accuracy; Pre, Precision; Se, Sensitivity/Recall.).

**Table 6:** Temporal Analysis of StressNet model with AlexNet and VGG-16 backbones, where N represent images of dataset of N days.

N		AlexNet				VGG-16		
	Tr. Loss	Val. Acc.	Pre	Se	Tr. Loss	Val. Acc	Pre	Se
3	0.5523	95.6522	0.9111	0.9111	0.5517	86.9525	0.8055	0.7603
6	0.5517	95.6522	0.9107	0.9333	0.5660	95.6522	0.8555	0.8079
9	0.5519	82.6087	0.8498	0.7523	0.5516	95.6522	0.8484	0.7904
11	0.5517	82.6087	0.8296	0.7746	0.6051	73.913	0.5726	0.5587
13	0.5619	82.6087	0.7888	0.7888	0.5515	91.3043	0.8888	0.8857

(Tr. Loss, Training loss; Val. Acc., Validation Accuracy; Pre, Precision; Se, Sensitivity/Recall).

**Table 7:** Class-level accuracy of the best StressNet model.

Class Name	Class Label	Accuracy Score
I1N2	0	0.900
I2N2	1	1.000
I3N2	2	0.833

**Table 8:** Classification report of the best StressNet model.

Class	Precision	Recall	F1-Score	Support
0	0.90	0.90	0.90	20
1	1.00	1.00	1.00	14
2	0.83	0.83	0.83	12

### 3.2 Computational complexity

The best model (with the VGG-16 backbone) took 75 minutes to train for 100 epochs. The model consists of 14,060,611 parameters that include both trainable parameters (weights and biases) and non-trainable parameters. Considering that each parameter is stored as a 64-bit floating-point value, the estimated memory consumption of the proposed model is around 107.274 megabytes.

### 3.3 Ablation study

We performed an ablation study to assess the impact of temporal and spectral data on the proposed model’s performance. This involved systematically reducing the number of temporal data used and spectral channels. Additionally, the study investigates the influence of the deformable convolution layer in comparison to standard convolution operation, along with the use of a BiLSTM network with weighted attention. These experiments aim to provide comprehensive evidence supporting the efficiency of our proposed method. The analysis includes the following cases.

1. Case I: Standard Convolution with BiLSTM.
2. Case II: Standard Convolution with BiLSTM and Weighted Attention.
3. Case III: Deformable Convolution with BiLSTM.

## 4 Discussion

For Spectral analysis, from **Table 5**, it can be inferred that our proposed model with AlexNet backbone achieves highest validation accuracy of 86.96% when using RGB-NIR channels as NIR band is good at highlighting the edges. With VGG-16 backbone, validation Accuracy is lowest of 65.22% when just using RGB bands. The addition of NIR and Re channels significantly increases accuracy and also with improvement in precision and sensitivity. The model’s performance is highest when using all spectral channels. In summary, for AlexNet, the addition of NIR channels significantly improves performance, while for VGG-16, the inclusion of all channels, particularly RGB-NIR-Re, yields the highest performance. Both models benefit from the inclusion of multiple spectral channels, with VGG-16 (best model) showing higher overall accuracy and performance. In the temporal analysis, as shown in **Table 6**, our proposed model with the AlexNet backbone demonstrates strong performance with 3 and 6 days of data, achieving a high accuracy of 95.65%. Although there is a slight decrease in precision, sensitivity improves. However, when the number of temporal data increases, the model’s performance drops to 82.60%, accompanied by a notable decrease in precision and sensitivity. On the other hand, our proposed model with the VGG-16 backbone exhibits a gradual increase in validation accuracy, going from 86.95% with 3 days of data to 95.65% with 9 days’ data. However, there is a performance decrease when using 11 days of data. Notably, the model performs exceptionally well with 13 days of data, achieving a validation accuracy of 91.30% along with improved precision and recall. It’s worth highlighting that this model achieves 95.65% validation accuracy using only 6 days of data, indicating the potential for early identification of water-stressed crops.

**Figure 6:**
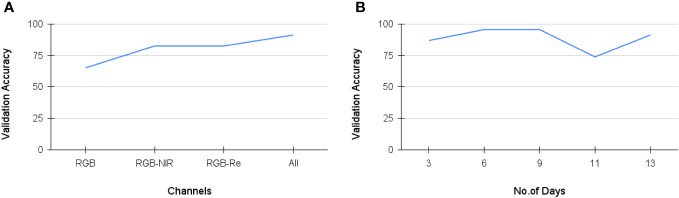
**(A)** Validation accuracy of best StressNet model with respect to Spectral Analysis; **(B)** Validation accuracy of best StressNet model with respect to Temporal Analysis.

From **Figure 6A**, it is evident that the performance of the best model (StressNet with VGG-16 backbone) gradually improves with the addition of NIR and Re spectral bands alongside RGB bands, signifying that incorporating both red-edge and NIR channels enhances the model’s capability. **Figure 6B** illustrates a progressive increase in the model’s performance up to 9 days. Subsequently, there is a decrease in performance between days 9 and 11, followed by an increase again.

### 4.1 Spectral analysis

In the spectral analysis conducted as part of the ablation study, three experiments were considered: RGB, RGB+NIR, RGB+Re, and all bands (as shown in **Table 9**). In Case I, the VGG-16 model achieved the highest test accuracy of 95.65% using RGB and red-edge data, highlighting the significance of spectral information for model robustness. In Case II, the VGG-16 model achieved the highest test accuracy of 95.65% when using all spectral bands. In Case III, the AlexNet model achieved the highest accuracy of 91.30% with RGB and red-edge information. Notably, the model a precision of 0.9027 (as shown in Case I) with standard convolution using RGB and Re bands. In Case II, with standard convolution and the integration of the BiLSTM network and weighted attention, the VGG-16 backbone model achieved a precision of 0.8727. In Case III, when using deformable convolutional layer with BiLSTM and weighted attention, along with AlexNet as the backbone, the model achieved a precision of 0.9047 with RGB and red-edge information. However, in cases where VGG-16 served as the backbone, the NIR and Re bands introduced essential features, leading the deformable convolutional layer to capture redundant spatial feature vectors and ultimately resulting in a reduction in accuracy compared to RGB data.

**Table 9:** Spectral Analysis. Case-I: Feature Extractor with BiLSTM network, Case-II: Feature Extractor with BiLSTM network and Weighted Attention, Case-III: Feature Extractor with Deformable Convolution and BiLSTM network.

Case	Feature Extractor	Metric	RGB	RGB-NIR	RGB-Re	All
***Case - I***	**AlexNet**	Tr. Loss	0.5551	0.5543	0.5546	0.5534
		Val. Acc.	91.3043	91.3043	82.6087	86.9565
		Pre	0.9	0.9444	0.62	0.83
		Se	0.79	0.8968	0.56	0.8
	**VGG - 16**	Tr. Loss	0.883	0.5536	0.5785	0.5729
		Val. Acc.	82.6087	65.2174	**95.6522**	86.9565
		Pre	0.5087	0.6809	0.9027	0.856
		Se	0.5238	0.6015	0.8333	0.8238
***Case - II***	**AlexNet**	Tr. Loss	0.5527	0.5522	0.5532	0.5525
		Val. Acc.	56.5217	82.6087	56.5217	78.2609
		Pre	0.4583	0.7269	0.4814	0.7416
		Se	0.4539	0.7269	0.466	0.7349
	**VGG - 16**	Tr. Loss	1.0693	0.5717	0.562	0.5627
		Val. Acc.	43.4783	91.3043	82.6087	**95.6522**
		Pre	0.1449	0.8727	0.7051	0.787
		Se	0.3333	0.8555	0.6634	0.7968
***Case - III***	**AlexNet**	Tr. Loss	0.5537	0.5534	0.5533	0.5517
		Val. Acc.	78.2609	82.6087	**91.3043**	78.2609
		Pre	0.6428	0.8214	0.9047	0.8333
		Se	0.6079	0.738	0.7936	0.7666
	**VGG - 16**	Tr. Loss	0.5515	0.6397	0.5877	0.6125
		Val. Acc.	82.6087	78.2609	69.5652	78.2609
		Pre	0.7306	0.7348	0.5958	0.6888
		Se	0.7111	0.6873	0.5539	0.673

Tr. Loss, Training Loss; Val. Acc., Validation Accuracy; Pre, Precision; Se, Sensitivity.

### 4.2 Temporal analysis

In addition to spectral analysis, we conducted a temporal study, exploring various temporal windows ranging from 3 to 13 days (as shown in **Table 10**). In Case I, AlexNet model achieved the highest validation accuracy of 91.30% with three days of data. In Case II, VGG-16 model achieved the highest validation accuracy of 95.65% with nine days of data. In Case III, AlexNet model achieved the highest validation accuracy of 95.65% with six days of data. By introducing a deformable convolutional layer with six days of data, the accuracy increased to 95% from the 90% observed in Case I (Feature extractor + BiLSTM). In contrast, VGG-16 extracted more refined features were with nine days of data, capturing distinct water stress patterns. However, after that point, there was minimal change in accuracy. The test accuracy reached 95%, underscoring the significance of incorporating a weighted attention module. Nevertheless, the test accuracy dropped from 95% to 65% with the addition of deformable convolution, indicating that the deformable convolutional layer introduced unnecessary complexity and increased parameters, leading to overfitting.

**Table 10:** Temporal Analysis. Case-I: Feature Extractor with BiLSTM network, Case-II: Feature Extractor with BiLSTM network and Weighted Attention, Case-III: Feature Extractor with Deformable Convolution and BiLSTM network.

Case	Feature Extractor	Metric	3	6	9	11	13
***Case - I***	AlexNet	Tr. Loss	0.5548	0.5746	0.5541	0.5532	0.5534
		Val. Acc.	**91.3043**	86.9565	86.9565	73.913	86.9565
		Pre	0.8714	0.8517	0.8634	0.7724	0.83
		Se	0.8634	0.8634	0.8634	0.6492	0.81
	VGG - 16	Tr. Loss	0.5619	1.0689	0.5625	1.069	1.069
		Val. Acc.	65.2174	56.5217	82.6087	43.4783	43.4783
		Pre	0.5444	0.1449	0.7571	0.1449	0.1449
		Se	0.5698	0.3333	0.7412	0.3333	0.3333
***Case - II***	AlexNet	Tr. Loss	0.5533	0.5535	0.5524	0.5539	0.5527
		Val. Acc.	78.2609	90	82.6087	65.2174	56.5217
		Pre	0.7248	0.9696	0.7471	0.7361	0.4583
		Se	0.7269	0.9444	0.7269	0.5079	0.4539
	VGG - 16	Tr. Loss	0.5724	0.5572	0.552	0.552	0.5621
		Val. Acc.	73.913	86.9565	**95.6522**	95.6522	95.6522
		Pre	0.3552	0.7962	0.863	0.7833	0.6974
		Se	0.4523	0.5222	0.8777	0.7761	0.6571
***Case - III***	AlexNet	Tr. Loss	0.5529	0.5325	0.5529	0.5531	0.5517
		Val. Acc.	82.6087	**95.6522**	86.9565	82.6087	78.2609
		Pre	0.7458	0.9696	0.744	0.75	0.8333
		Se	0.6222	0.9444	0.7555	0.7555	0.7666
	VGG - 16	Tr. Loss	0.7858	0.5954	0.5795	1.0695	0.5515
		Val. Acc.	78.2609	78.2609	65.2174	43.4783	82.6087
		Pre	0.6388	0.7727	0.3789	0.1449	0.7306
		Se	0.6253	0.6492	0.4904	0.3333	0.7111

Tr. Loss, Training Loss; Val. Acc., Validation Accuracy; Pre, Precision; Se, Sensitivity.

The bold values highlighted highest validation accuracies obtained in that specific case.

### 4.3 Impact of deformable convolution

To assess the impact of deformable convolution, we examined Cases II and III in the ablation study (**Tables 9**, **10**). In the spectral analysis experiment, the AlexNet model’s performance increased from 56.52% validation accuracy to 78.26% with RGB bands. However, there was no change with RGB-NIR. Notably, with RGB-Re bands, the AlexNet model’s accuracy surged to 91.30%. For the VGG-16 model, adding the deformable convolutional layer with RGB bands raised the validation accuracy to 82.60% from 43.47%. However, introducing additional spectral channels led to a 10-20% drop in validation accuracy, likely due to increased model complexity, overfitting, and feature redundancy. Regarding temporal analysis, the AlexNet model achieved its highest validation accuracy of 90% with 6 days’ data. The model’s performance gradually declined as the number of days increased. In contrast, the VGG-16 model’s performance was more variable, reaching a peak of 82.60% (as shown in Case III). This suggests that deformable convolution enhances the extraction of spatial features, resulting in a richer vector representation across timestamps. As data increased from 3 to 6 days, the model’s performance exhibited a decreasing trend, suggesting a potential absence of identified geometrical transformations. The introduction of the deformable convolution layer added unnecessary complexity and increased the number of parameters, resulting in overfitting.

### 4.4 Impact of weighted attention based BiLSTM

To assess the impact of deformable convolution, we investigated Cases I and II in the ablation study (**Tables 9**, **10**). In the spectral analysis experiment, the AlexNet model achieved an impressive 91.30% validation accuracy. However, there was no significant improvement in performance when either NIR or Re channels were added. This limitation can be attributed to complex background variations in the data, which challenged the limited feature representation capacity of the AlexNet model, making it challenging to distinguish foreground information. In contrast, the VGG-16 model, with its deeper layers and the support of the BiLSTM network and weighted attention mechanism, effectively addressed complex backgrounds, resulting in a substantial performance increase from 86.95% to 95.65%. In the context of temporal analysis, the performance of the AlexNet model exhibited an initial increase, followed by a subsequent decrease as the data extended from 3 days to 9 days (as demonstrated in Case I). Beyond the 9th day, this pattern persisted. A similar trend was observed after introducing weighted attention (Case II). In contrast, the VGG-16 model demonstrated higher performance in both Case I and II up to 9 days, indicating the model’s resilience in managing temporal variations in images corresponding to the crop’s growth over time. Beyond this point, the performance remained relatively constant with 11 and 13 days’ data, suggesting negligible growth in the crops.

## 5 Conclusion

In this article, we propose a novel DL-based model titled StressNet, which aims to monitor water stress, especially in maize crop. StressNet consists of two key components, the first being CNN with a deformable convolutional layer, and the second is a BiLSTM network with weighted attention. The effectiveness of our framework is extensively validated through a comprehensive study utilizing multispectral and multi-temporal imagery captured by UAV. The best model achieved a validation accuracy of 91.30% with a training loss of 0.555. However, it is essential to acknowledge that our proposed method is validated using a dataset acquired from a controlled environment. However, the real-world scenario introduces more complexities. In such circumstances, it is essential to consider additional factors such as super-resolution, noise reduction, and plant shoot segmentation techniques. We will develop a DL pipeline with further additions in our future research. We encourage researchers to verify our findings using their datasets and expand upon our pipeline.

## Data availability statement

The raw data supporting the conclusions of this article will be made available by the authors, without undue reservation.

## Author contributions

TN, KK, and SC worked on the conceptualization and methodology of the paper; TN worked on data curation, generation and pre-processing. TN designed the experiments. KK and SC developed the code for the experiments. TN and KK conducted experiments and validated. TN wrote the original manuscript. TN, KK, and SC analyzed the findings and suggested the modifications in the manuscript. RP reviewed and supervised the work. RP, BN, and UD provided funding and resources for experimental site setup and data collection. All authors contributed to the article and approved the submitted version.
