# Supplementary figures and images for: StressNet: a spatial-spectral-temporal deformable attention-based framework for water stress classification in maize
Source: Front Plant Sci. 2023 Nov 28;14:1241921. doi: 10.3389/fpls.2023.1241921 (PMC10714940; doi:10.3389/fpls.2023.1241921)

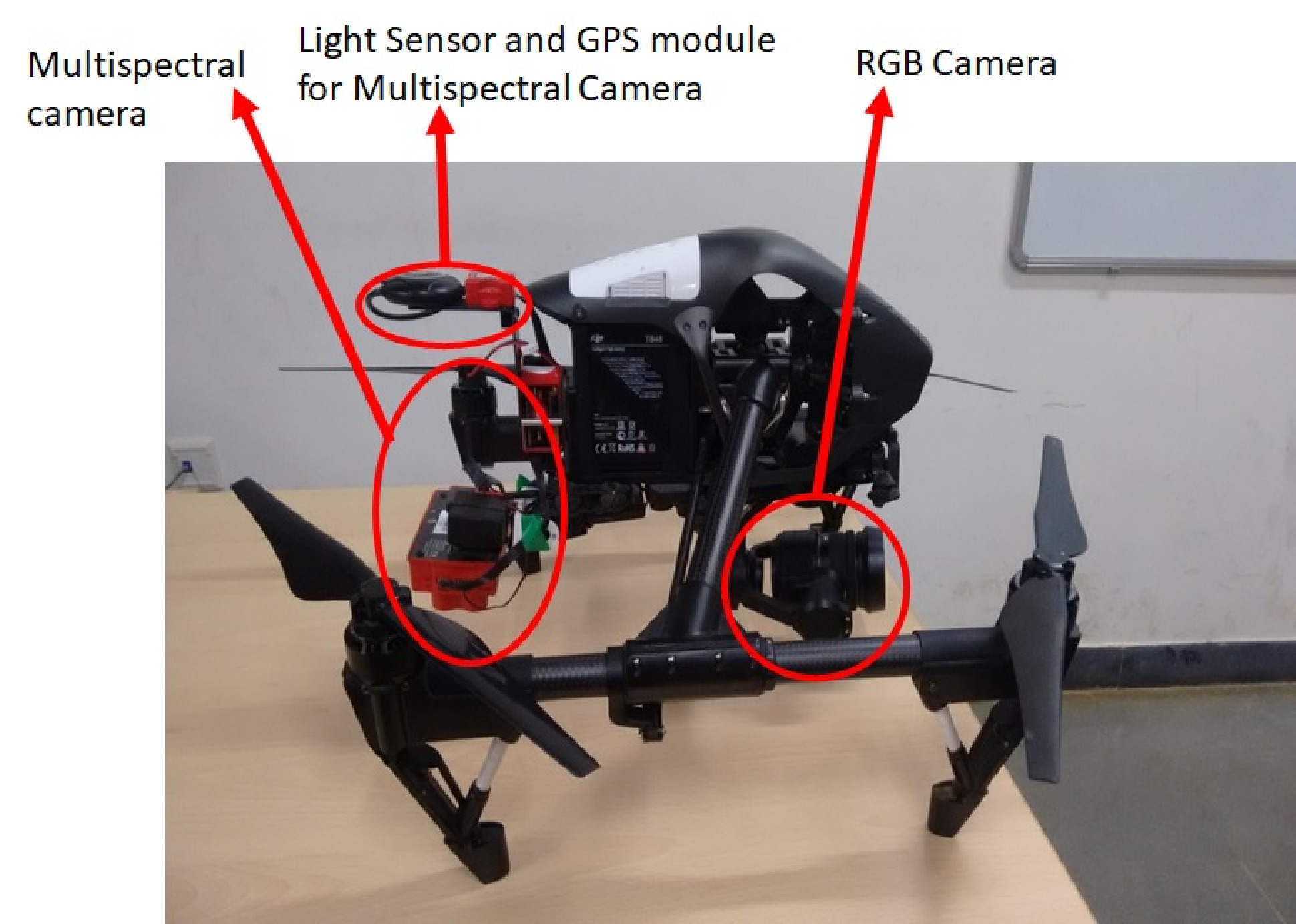

Supplement: Supplementary file 1 [file Image_1.jpeg]

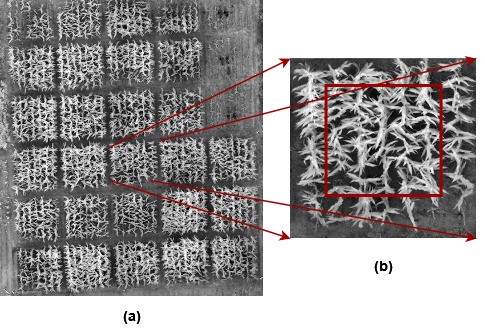

Supplement: Supplementary file 2 [file Image_2.jpeg]
